# On the analyses of carbon atom diffused into grey cast iron during carburisation process

**DOI:** 10.1038/s41598-022-22136-w

**Published:** 2022-10-31

**Authors:** Enesi Y. Salawu, Adeolu A. Adediran, Oluseyi O. Ajayi, Anthony O. Inegbenebor, Joseph O. Dirisu

**Affiliations:** 1 Department of Mechanical Engineering, Covenant University, P.M.B 1023, Ota, Ogun State Nigeria; 2 Department of Mechanical Engineering, Landmark University, P.M.B 1001, Omu-Aran, Kwara State Nigeria; 3 Department of Mechanical Engineering Science, University of Johannesburg, Johannesburg, South Africa

**Keywords:** Energy science and technology, Engineering, Materials science

## Abstract

The study employed Fick’s second law of diffusion to discover some unknown aspect of carbon diffusion in grey cast iron during carburisation process. Emphasis on the experiments and theoretical modelling were established for better accomplishments. Pulverised palm kernel and eggshell additives of 70 (wt.%) and 30 (wt.%) according to the Voige law of mixture was considered as a continuous medium without considering the atomic nature of the mixture. Furthermore, a kinetic approach was described where a physical model of the substrate immersed in the carbon mixture was established while diffusion equations were modelled to establish the mechanism of carbon diffusion during carburisation. Initial composition and concentration of diffused atom remained constant which are 2.68 and 6.67% carbon. While the carburizing time used varied from 60 min, 90 min, 120 min, 150 min, 180 min and 210 min respectively at constant carburising temperature of 900° The results revealed varying composition gradient of carbon atom ranging from 5.4%, 5.42%, 5.44%, 5.46%, 5.51%, and 5.65 compared to the initial carbon content of 2.68%. The concentration of carbon atom on the substrate surface at varying time implies that the process was non-steady state diffusion which verified Fick’s second law of diffusion. Hence, the composition achieved is a function of boundary conditions such as time position and temperature. This novel study will enhance the understanding of heat treat treatment of metals such that their applications in the industry will be numerous.

## Introduction

Metallic materials which have undergone heat treatment via carburisation process have surfaces characterised with improved mechanical properties^1^. They are basically modified for advanced engineering applications using diffusion mechanism^2^. Diffusion involved the squeezing of carbon atoms past its surrounding atoms in other to reach a new position. Diffusion process can be best understood from the equation parameter of Fick’s law as well as the knowledge of the activation energy required for the diffusion process^3^. For instance, Fick’s second law established a non-steady state diffusion of atoms as described by the differential equation $\frac{dc}{{dt}} = \frac{{Dd^{2} }}{{dx^{2} }}$ of which the solution is a function of a particular diffusion process described by the boundary parameters in the Eq. 1^4^.

1 $$ \frac{{C_{s} - C_{x} }}{{C_{s} - C_{0} }} = erf\left( {\frac{x}{{\sqrt[2]{Dt}}}} \right) $$

The solution to Fick’s second law permits the evaluation of concentration of an atom diffused near the surface of the coupon material as a function of time and distance, provided that the coefficient of diffusion D remains constant and the concentration of the atom at the surface $C_{s}$ as well as within the material $C_{0}$ remain unchanged^5^. Recent study on the diffusion of palm kernel and eggshell additives to grey cast iron resulted in increase in the hardness of the material^6^. The tribological properties of the treated material via diffusion process was excellent which made it suitable for advanced engineering material^7,8^. The principle of Fick’s second law had been limitedly used in evaluating the depth of the mechanical properties that had been diffused into these materials, thus making the analysis or establishing the statistical significance of the diffused carbon atom a major problem^9–11^. One major problem in diffusion analysis is the determination of the temperature field and depth of carbon imposed on the surroundings of the substrate metal^12,13^. Study has shown that the knowledge of the temperature distribution could be a pointer to understanding the carbon diffusion mechanism as well as the depth of diffusion^14^. For grey cast iron material, knowledge of the carbon diffusion is important in analysing the structural integrity. Also, knowledge of carbon diffusion is critical to the optimisation of the coating thickness as well as the compatibility of the carburising agents^15–20^. However, movement of atoms is an essential factor for the diffusion process to take place in metals. Therefore, understanding the dynamics of the diffusion process remain a crucial problem in the determination of the carbon depth in solid materials^21^.

Furthermore, carbon content such as that from palm kernel, coconut, wood charcoal and eggshells have been the major carbon content that are usually deployed during carburisation process. The eco-friendliness and their environmental friendliness make them most promising materials for advanced heat treatment applications. However, the commercial applications of these organic materials are limited by some drawbacks including the determination of the depth of carbon diffusion^22^. To address these problems and improve on the applications of these organic materials, various material, methods and concepts have been designed and developed. For instance, Chen et al.^23^ developed a light-weight thermal energy concrete and reinforced it with carbon from palm kernel shell. This helped in improving the thermal lag and lowered the peak temperature as a composite. More so, alloying of steel with borides under high temperature resulted in increase in the hardness of the substrate^24^ and the thickness of the layers increased with increased temperature as reported by Hu et al.^25^. It has also been reported that carbon diffuses into the surface and settles at the face centred cubic region during the carburisation of austenitic steel. This therefore, caused an increase in the hardness of the layers formed and leaving chromium in its free form and allows an increase in corrosion resistance as well as improvements in tribological and mechanical properties. However, it is important to understand that in an ideal diffusion process, a diffusing atom squeezes past the surrounding atoms to get to the new position. This implies that energy will be utilized to force the atom to its new position. Thus, the energy barrier needed to move the atom to its new position is referred to as the activation energy^26–30^.

In our present research, diffusion in grey cast iron using pulverised palm kernel and eggshell additives showed an increase in the hardness of the substrate material. Therefore, the aim of this study is to deploy the Fick’s Second Law in the determination of the depth of carbon in the material that resulted in increased hardness after the carburisation process. This method had become prominent in the understanding of diffusion in solids, liquids and gases.

## Experimental details

### Carburisation process

The experimental approach involved the use of pulverised palm kernel and eggshell additives of 70 (wt.%) of pulverised palm kernel and 30 (wt.%) of pulverised eggshells according to the Voige law of mixture. Grey cast iron substrates of dimensions (20 mm × 20 mm × 10 mm) and chemical composition (wt.%) of 2.68 C, 1.42 Si, 0.63 Mn, 0.13 S, 0.28 P was prepared using different grades of silicon carbide abrasives to obtain a polished and smooth surface for easy carbon diffusion. Prepared grey cast iron substrate were embedded into some stainless steel containers to reduce the rate of carbon absorption and were finally charged into a muffle furnace of 1200 ℃ capacity. The carburisation process was carried out at a temperature of 900 ℃ for 60, 90,120, 150, and 180 min after which it was stopped and cooling was done using water. Water was used as a quenching medium due to its natural convective heat transfer ability and the tendency of increasing the hardness property of the substrate. Initial composition and concentration of diffused atom remained constant which are 2.68 and 6.67%. This is to be able to determine the percentage of carbon atom diffused into the material surface, depth of diffusion at a particular time.

### Heat transfer analyses

The main aim of this analyses is to determine the temperature field impacted by the surrounding medium (carburisers) on the carburised material (grey cast iron). Since, conduction process takes place for proper diffusion of carbon into the material, it is important to understand and analyse the temperature distribution around the material. To achieve this, Fourier’s law of heat conduction was employed.

**Figure 1:**
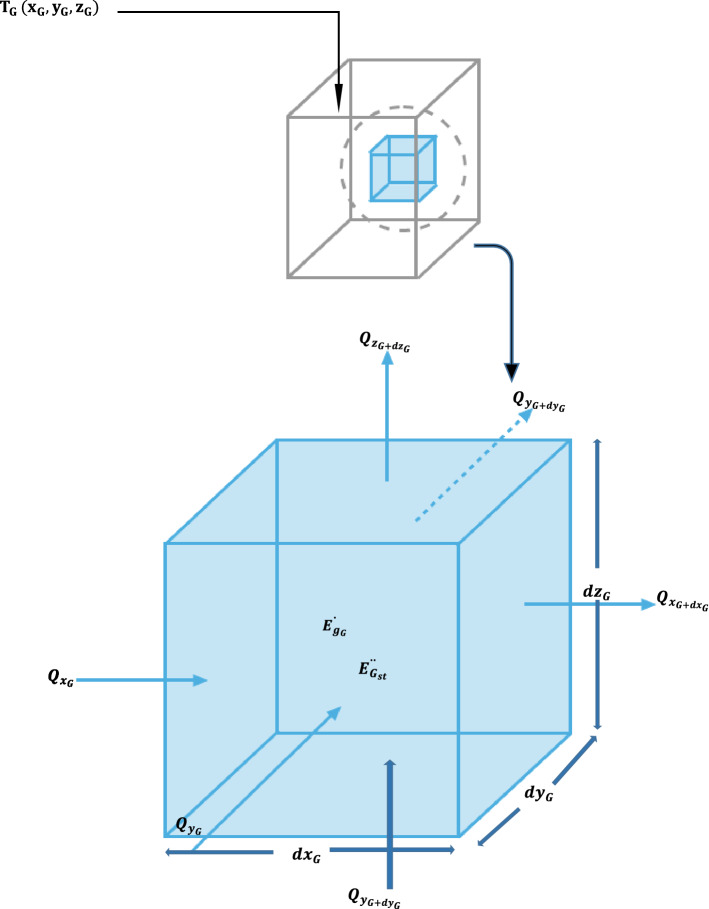
Conduction analysis in Cartesian coordinate.

Consider a grey cast iron substrates of dimensions (20 mm × 20 mm × 10 mm) immersed in a homogenous medium in which there is no bulk motion as demonstrated in Fig. 1. Let the temperature distribution be described by a Cartesian coordinates in the form, $T_{G } \left( {x_{G} , y_{G} , z_{G} } \right)$. Assuming an infinitesimally control volume defined by $dx_{G} \times dy_{G } \times dz_{G}$. Conduction heat transfer occurred at each control surface due to temperature variation, therefore the rate of heat conducted perpendicular to the coordinates $x_{G} - , y_{G} - , z_{G}$ can be denoted by $Q_{{x_{G} }} ,\;Q_{{y_{G} }} ,\;Q_{{z_{G} }}$ respectively.

Therefore, the rate of heat conduction is given by the equations;

2 $$ Q_{{x_{G} }} - Q_{{x_{{G + dx_{G} }} }} = - \frac{{\partial Q_{{x_{G} }} }}{{\partial x_{G} }}dx_{G} \Rightarrow Q_{{x_{{G + dx_{G} }} }} = Q_{{x_{G} }} + \frac{{\partial x_{G} }}{{\partial x_{G} }}dx_{G} $$

3 $$ Q_{{y_{G} }} - Q_{{y_{{G + dy_{G} }} }} = - \frac{{\partial Q_{{y_{G} }} }}{{\partial y_{G} }}dy_{G} \Rightarrow Q_{{y_{{G + dy_{G} }} }} = Q_{{y_{G} }} + \frac{{\partial y_{G} }}{{\partial y_{G} }}dy_{G} $$

4 $$ Q_{{z_{G} }} - Q_{{z_{{G + dz_{G} }} }} = - \frac{{\partial Q_{{z_{G} }} }}{{\partial z_{G} }}dz_{G} \Rightarrow Q_{{z_{{G + dz_{G} }} }} = Q_{{z_{G} }} + \frac{{\partial z_{G} }}{{\partial z_{G} }}dz_{G} $$

The thermal energy generated within the medium is given by;

5 $$ \dot{E}_{{g_{G} }} = \dot{Q}_{G} \times dx_{G} \times dy_{G } \times dz_{G} $$

Based on the thermal energy generated, there was variation in the internal thermal energy stored by the grey cast iron material which is being carburised. However, in the absence of phase transformation, latent energy is minimal and the stored energy is given by;

6 $$ \ddot{E}_{{G_{st} }} = \rho c_{p} \frac{\partial T}{{\partial t}}\left( {dx_{G} \times dy_{G } \times dz_{G} } \right) $$

The conservation energy required within the medium is given by;

7 $$ \ddot{E}_{{G_{st} }} = \dot{E}_{{in_{G} }} + \dot{E}_{{g_{G} }} - \dot{E}_{{out_{G} }} $$

Since the rate of heat conduction involve energy input and output and substituting Eqs. 5 and 6 into Eq. 7 yield

8 $$ \rho c_{p} \frac{\partial T}{{\partial t}}\left( {dx_{G} \times dy_{G } \times dz_{G} } \right) = Q_{{x_{G} }} + Q_{{y_{G} }} + Q_{{z_{G} }} + \dot{Q}_{G} dx_{G} dy_{G } dz_{G} - \left( {Q_{{x_{{G + dx_{G} }} }} + Q_{{y_{{G + dy_{G} }} }} + Q_{{z_{{G + dz_{G} }} }} } \right) $$

9 $$ \rho c_{p} \frac{\partial T}{{\partial t}}dx_{G} dy_{G } dz_{G} = Q_{{x_{G} }} + Q_{{y_{G} }} + Q_{{z_{G} }} + \dot{Q}_{G} dx_{G} dy_{G } dz_{G} - Q_{{x_{{G + dx_{G} }} }} - Q_{{y_{{G + dy_{G} }} }} - Q_{{z_{{G + dz_{G} }} }} $$

Substituting from Eqs. 2–4, into Eq. 9 yields;

10 $$ \rho c_{p} \frac{\partial T}{{\partial t}}dx_{G} dy_{G } dz_{G} = - \frac{{\partial Q_{{x_{G} }} }}{{\partial x_{G} }}dx_{G} - \frac{{\partial Q_{{y_{G} }} }}{{\partial y_{G} }}dy_{G} - \frac{{\partial Q_{{z_{G} }} }}{{\partial z_{G} }}dz_{G} + \dot{Q}_{G} dx_{G} dy_{G } dz_{G} $$

Assuming that the material become isotropic after the carburisation process, then the conduction heat rate can be established from Fourier’s law;

11 $$ Q_{{x_{G} }} = - K\frac{\partial T}{{\partial x_{G} }};\;Q_{{y_{G} }} = - K\frac{\partial T}{{\partial y_{G} }};\;Q_{{z_{G} }} = - K\frac{\partial T}{{\partial z_{G} }} $$

Each equation from Eq. 11 represents the heat flux resulting from diffusion across the substrate

12 $$ Q_{{x_{G} }} = - Kdy_{G } dz_{G} \frac{\partial T}{{\partial x_{G} }} $$

13 $$ Q_{{y_{G} }} = - Kdx_{G } dz_{G} \frac{\partial T}{{\partial y_{G} }} $$

14 $$ Q_{{z_{G} }} = - Kdx_{G } dy_{G} \frac{\partial T}{{\partial z_{G} }} $$

Substituting the heat flux Eqs. 11, 12, and 13 into Eq. 9 and dividing through the control volume $\left( {dx_{G} dy_{G } dz_{G} } \right)$, we obtain

15 $$ \frac{\partial }{{\partial x_{G} }}\left( {k\frac{\partial T}{{\partial x_{G} }}} \right) + \frac{\partial }{{\partial y_{G} }}\left( {k\frac{\partial T}{{\partial y_{G} }}} \right) + \frac{\partial }{{\partial z_{G} }}\left( {k\frac{\partial T}{{\partial z_{G} }}} \right) + \dot{Q}_{G} = \rho c_{p} \frac{\partial T}{{\partial t}}. $$

Equation 15 is the heat transfer equation when the substrate is represented in the Cartesian coordinate. This model establishes the fundamentals of the heat conducted through the substrate material. Thus, it is possible to obtain the temperature distributed during the carburisation process.

Assuming the temperature distribution across the substrate is ven by the equation;

16 $$ T\left( {X_{G} Y_{G} Z_{G} } \right) $$

This equation is expressed as a function of time and also establishes the energy conservation. Thus, $\frac{\partial }{{\partial x_{G} }}\left( {k\frac{\partial T}{{\partial x_{G} }}} \right)$ is related to the net heat conducted into the substrate in x-coordinate direction

17 $$ \frac{\partial }{{\partial x_{G} }}\left( {k\frac{\partial T}{{\partial x_{G} }}} \right)dx_{G} = Q_{{X_{G} }}^{n} - Q_{{x_{G} + dx_{G} }}^{n} $$

The same can be expressed in the y- and z-coordinates to obtain Eqs. 18 and 19

18 $$ \frac{\partial }{{\partial y_{G} }}\left( {k\frac{\partial T}{{\partial y_{G} }}} \right)dy_{G} = Q_{{y_{G} }}^{n} - Q_{{y_{G} + dy_{G} }}^{n} $$

19 $$ \frac{\partial }{{\partial z_{G} }}\left( {k\frac{\partial T}{{\partial z_{G} }}} \right)dz_{G} = Q_{{z_{G} }}^{n} - Q_{{z_{G} + dz_{G} }}^{n} $$

Therefore, for a constant thermal conductivity, Eq. 15 can be rewritten as;

20 $$ \frac{{\partial^{2} T}}{{\partial x_{G}^{2} }} + \frac{{\partial^{2} T}}{{\partial y_{G}^{2} }} + \frac{{\partial^{2} T}}{{\partial z_{G}^{2} }} + \frac{{\dot{Q}_{G} }}{K} = \frac{1}{\alpha }\frac{\partial T}{{\partial t}} $$

where $\alpha = \frac{K}{{\rho C_{p} }}$ is the thermal diffusivity.

### Depth of carbon deposited based on Fick’s Second Law

According to Fick’s Second law, carbon atom diffused during the carburisation can be defined by the differential equation in the form [32];

21 $$ \frac{dc}{{dt}} = D\frac{{d^{2} c}}{{dx^{2} }} $$

And the boundary conditions for the carburisation process depends on the equation [32];

22 $$ \frac{{c_{s} - c_{x} }}{{c_{s} - c_{0} }} = erf\left( {\frac{x}{{2\sqrt {Dt} }}} \right) $$

where $$c_{0}$$ the carbon content for the as-received grey cast iron which is given by 2.68%, $$c_{s}$$ assumed value from iron-carbon alloy system diagram between pure iron an interstitial compound, iron carbide (Fe_3_C), containing 6.67% carbon. $$c_{x}$$ Implies the concentration of the diffused carbon at a depth denoted by x in millimetre below the material surface at time t. From the carburisation result, the value for $$c_{x} = 5.40\%$$ at t = 60 min which is 3600 s, D is the diffusion coefficient and it remain constant for constant, $$c_{s}$$ provided $$c_{0} $$ also remain constant. D = 2 × 10^−11^ (m^2^ s^−1^) of carbon in FCC iron interstitial carbon diffusion [33].

Thus, based on these conditions, the solution to Fick’s second law enabled the study to determine the concentration of a diffused carbon atom as a function of carburising time and distance (depth). erf = error function which is given by 0.71 [34], t = time of carburisation which is given as 3600 s, x = is the depth (mm)?

Substituting the values into Eq. 22

$$ \begin{aligned} & \frac{6.67 - 5.40}{{6.67 - 2.68}}{ = } 0.71\left( {\frac{x}{{2\sqrt {\left( {2 \times 10^{ - 11} } \right)3600} }}} \right) \\ & {\text{x}} = 0.0{144}\;{\text{mm}} \\ \end{aligned} $$

## Results and discussion

### Diffusion of carbon atoms into the grey cast iron

**Figure 2:**
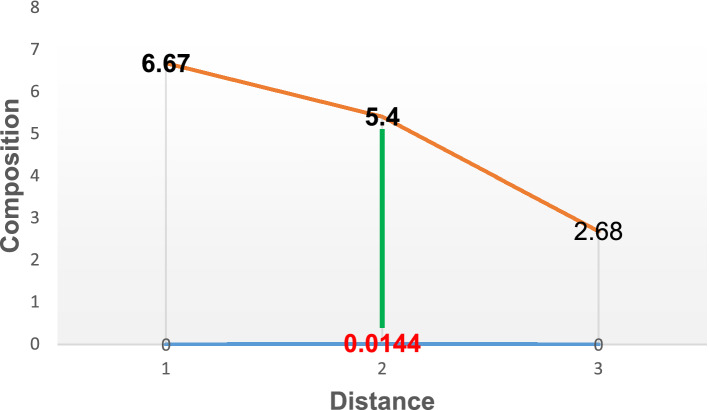
Diffusion of carbon atoms into the surface of grey cast iron at 900° for 60 min.

**Figure 3:**
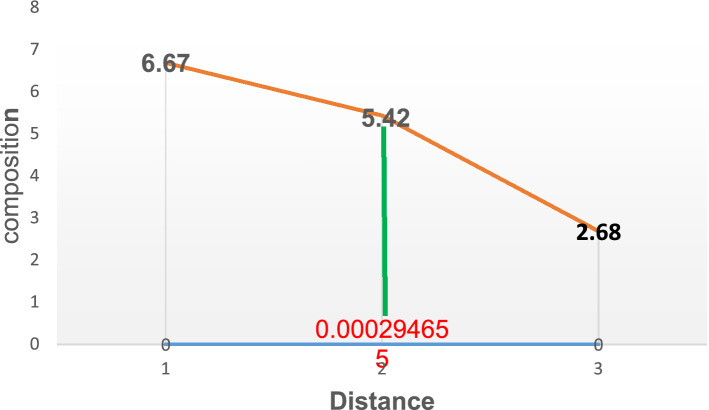
Diffusion of carbon atoms into the surface of grey cast iron at 900° for 90 min.

**Figure 4:**
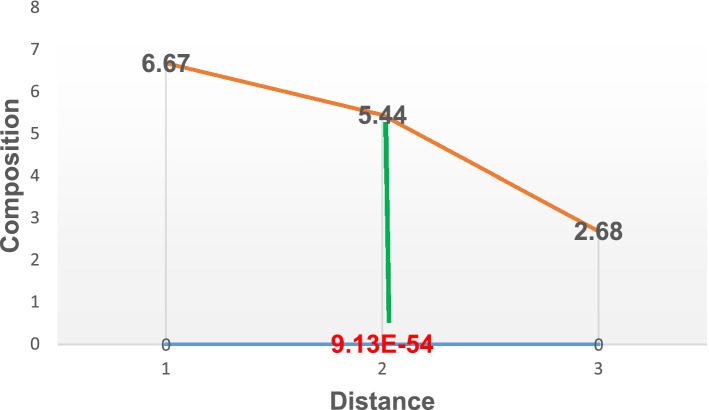
Diffusion of carbon atoms into the surface of grey cast iron at 900° for 120 min.

**Figure 5:**
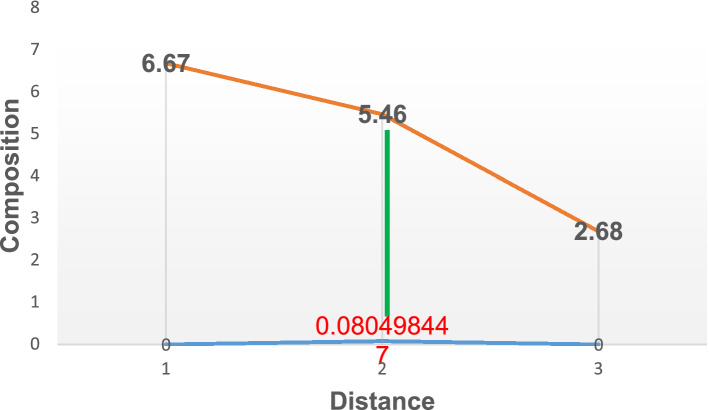
Diffusion of carbon atoms into the surface of grey cast iron at 900° for 150 min.

**Figure 6:**
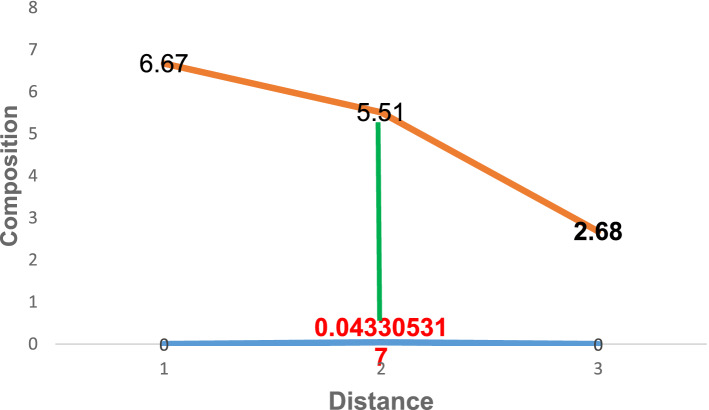
Diffusion of carbon atoms into the surface of grey cast iron at 900° for 180 min.

**Figure 7:**
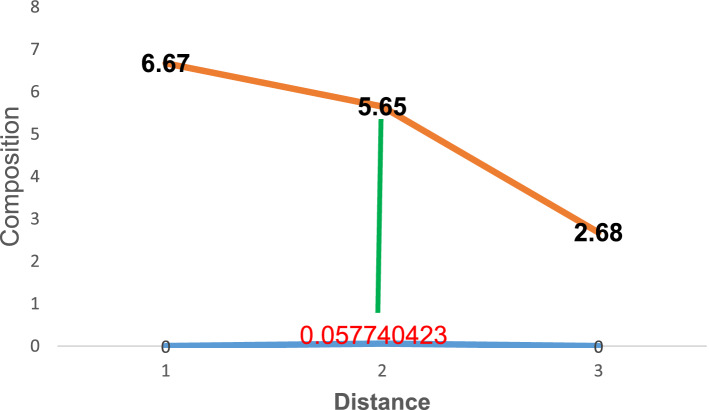
Diffusion of carbon atoms into the surface of grey cast iron at 900° for 210 min.

Figures 2, 3, 4, 5, 6 and 7 illustrate the variation in percentage composition of carbon atoms diffused at various time at a temperature of 900°. From Fig. 2, it was observed that 5.4% of carbon diffused into the material at a depth of 0.0144 mm at 60 min. Comparing this with the initial composition of 2.68%, there was an increase of about 2.72% in carbon content on the material surface. Thus, the driving force for carbon diffusion led to microstructural changes in the substrate carbon content.

In addition, Fig. 3 illustrates the carbon atom diffusion at 90 min. About 5.40% was deposited at a depth of 0.000294655 mm. about 0.02% increase compared to the diffusion at 60 min. Further to this, with the temperature of diffusion still kept at 900° while increasing the diffusion time (holding time) to 120 and 150 min resulted in increased composition in carbon atom to about5.44 and 5.46 respectively as described by Figs. 4 and 5. The corresponding depth of deposition into the material surface were 9.13E-54 and 0.080498 as well. Thus, the result established the fact that, increased diffusion time will lead to increase in carbon deposition as well.

Similarly, Figs. 6 and 7 depicts the composition of carbon atom at 180 and 210 min respectively at the same temperature of carburisation. The composition at this time were 5.51 and 5.65 respectively at a distance of 0.043305317 and 0.0577404231. In comparison with the initial carbon composition, it was observed that an increase of about 2.83 and 2.97 was added at various time of carburisation respectively. Thus, the solution to Fick’s second law permits the study to establish the composition of diffusing carbon atom near the substrate surface as a function of time and distance respectively.

Hence, carburisation was defined in this study as the diffusion of carbon into the substrate metal. The purpose was to determine the concentration gradient of carbon at varying time since the entire process involved thermal effects. Thus, the processing parameters such as temperature and time are the key influencers of diffused carbon potentials. From the results, it is noteworthy to say that irrespective of the interaction between the carbon and other alloying elements present in the carbon, diffusion of carbon into the substrate (grey cast iron) was a perfect description of Fick’s Second Law of diffusion. This is because the composition gradient of carbon atom near the surface of the substrate vary with time due to carbon accumulation. This is the reason why it is called a non-steady state diffusion as expressed in the modelled equations.

### Scanning electron microstructure of the carburised samples

**Figure 8:**
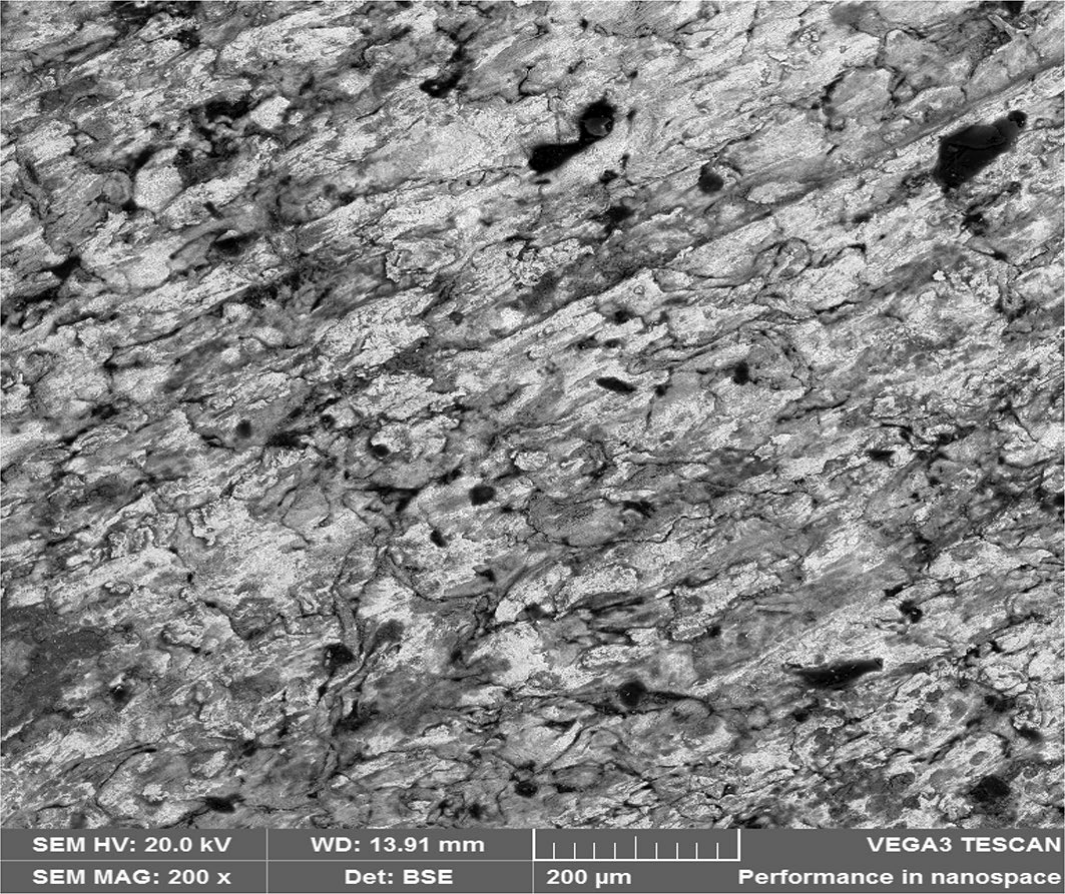
SEM microstructure of As-received grey cast iron.

**Figure 9:**
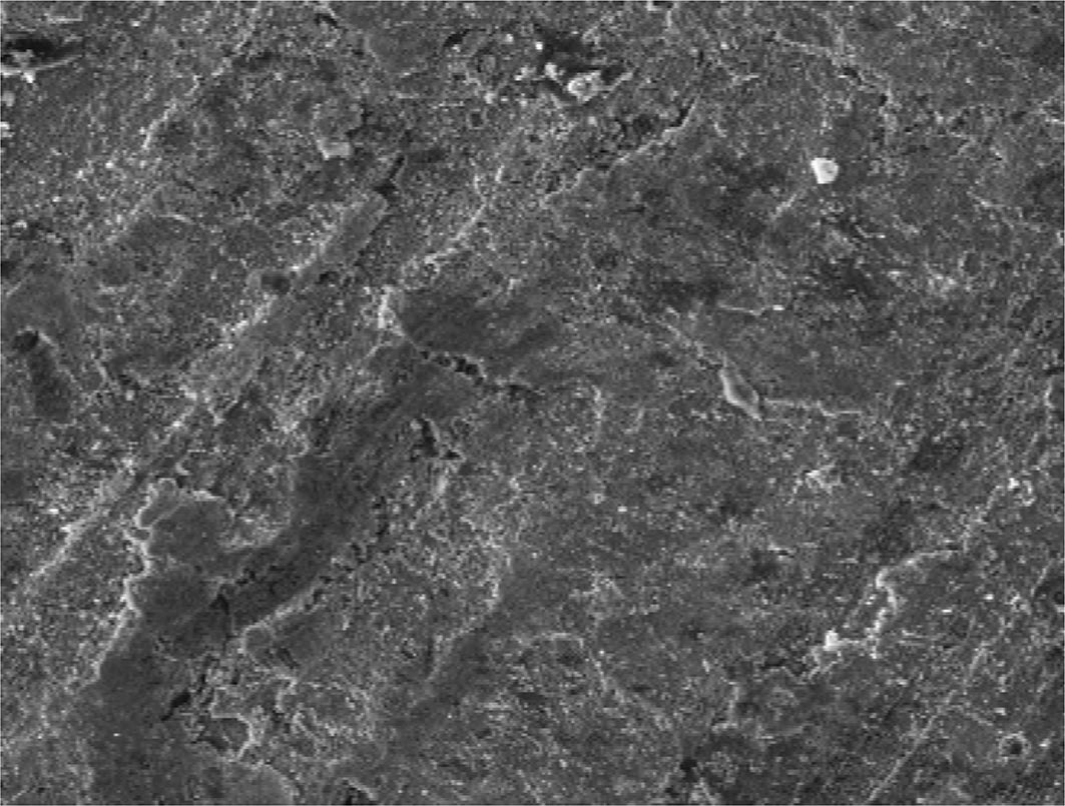
SEM microstructure of grey cast iron carburized at 900 °C for 60 min.

**Figure 10:**
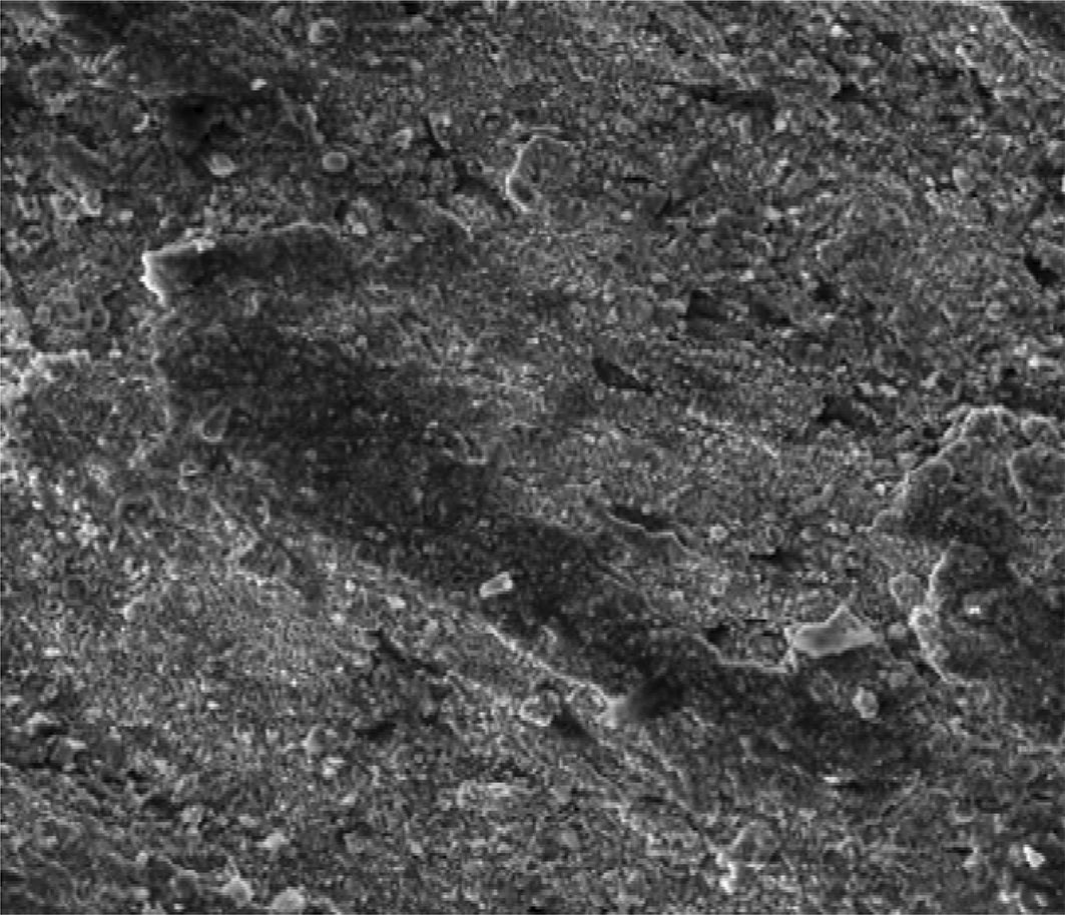
SEM microstructure of grey cast iron carburized at 900 °C for 90 min.

**Figure 11:**
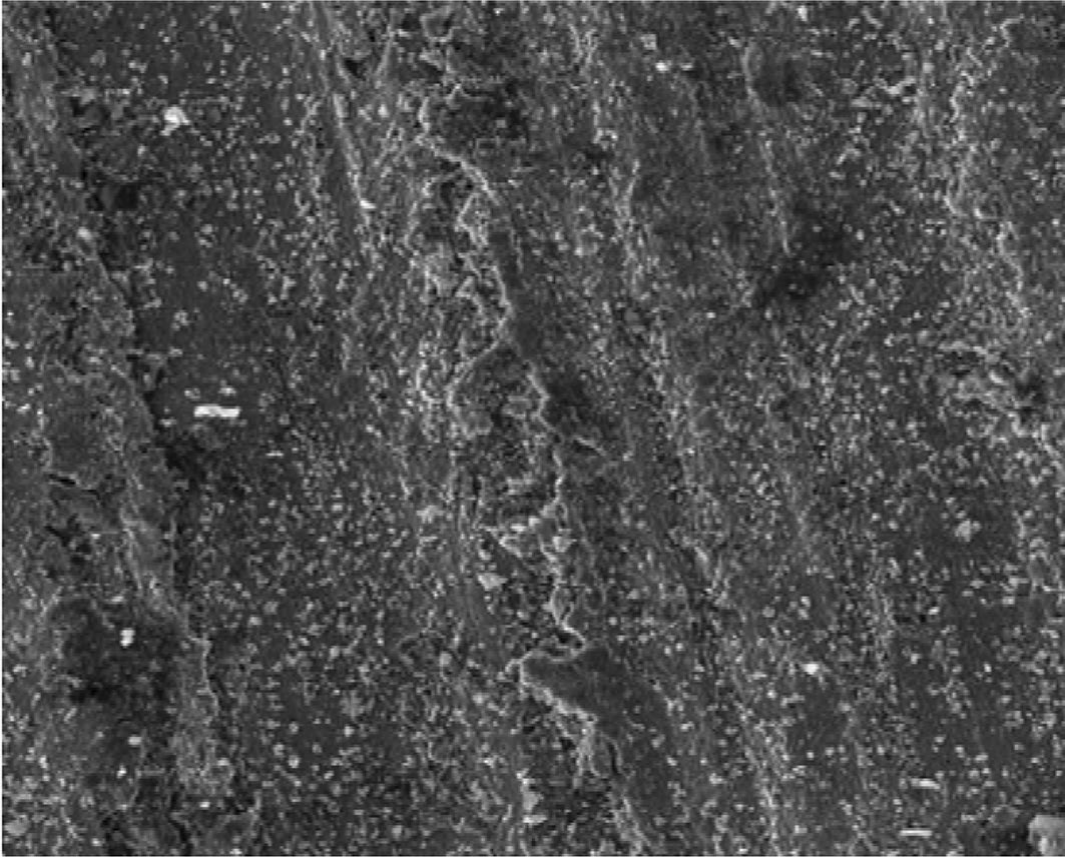
SEM microstructure of grey cast iron carburized at 900 °C for 120 min.

**Figure 12:**
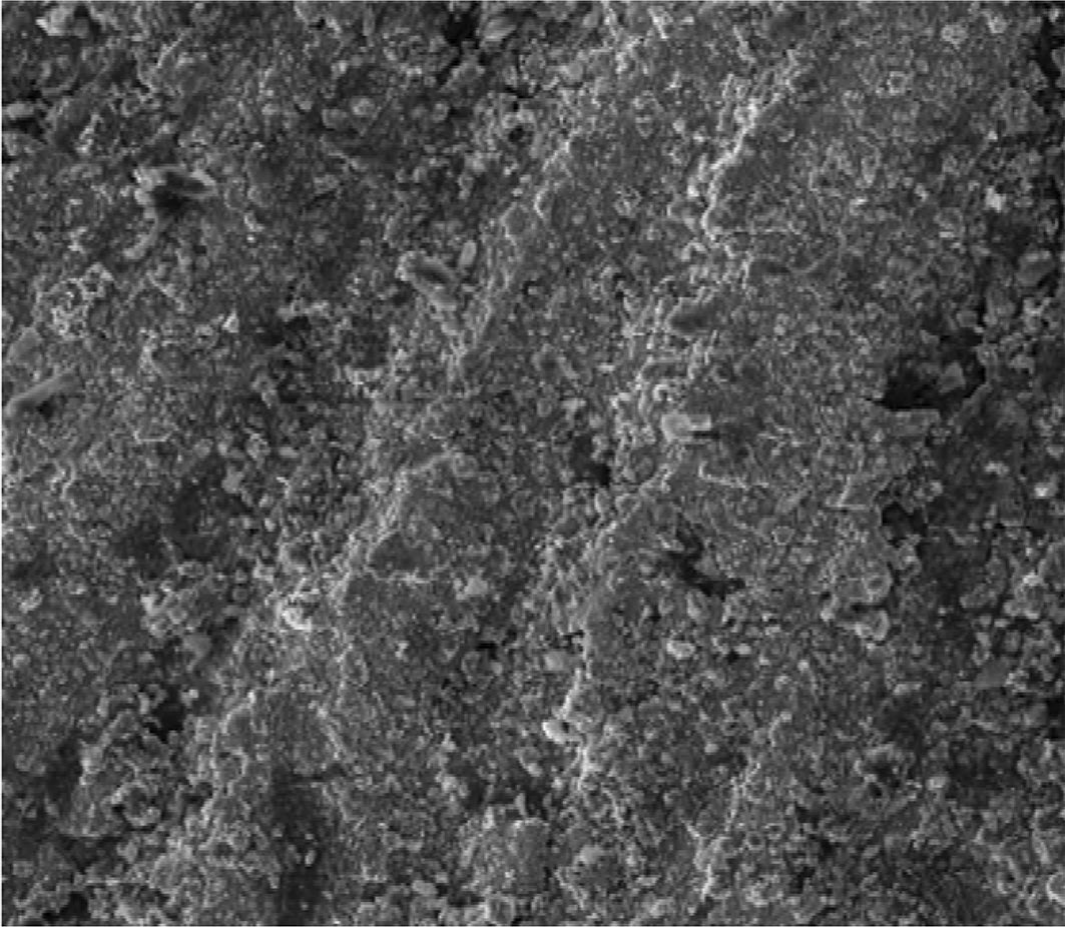
SEM microstructure of grey cast iron carburized at 900 °C for 150 min.

**Figure 13:**
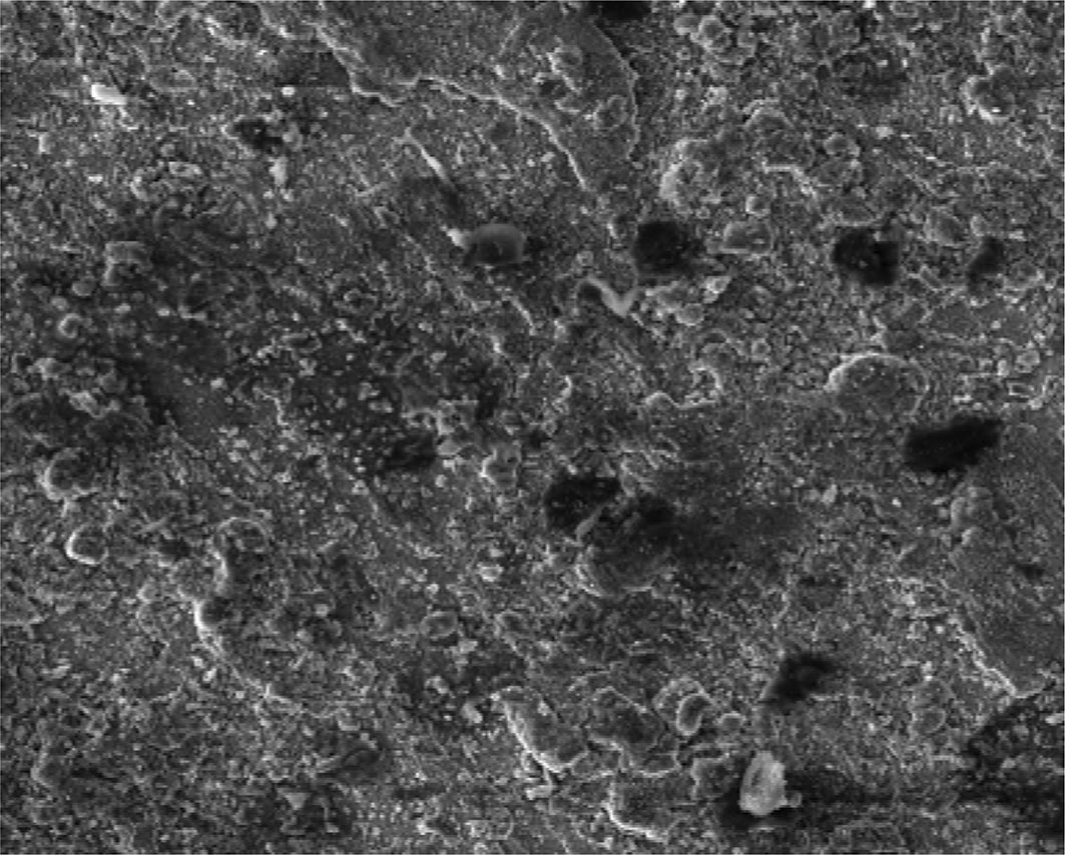
SEM microstructure of grey cast iron carburized at 900 °C for 180 min.

Figure 8 showed the SEM microstructure of the as-received grey cast iron before carburisation. Similarly, Fig. 9 showed the microstructure of grey cast iron carburised at 900 °C at 60 min. From the Figure, it was observed that graphite dominates the metal surface which is traceable to the nature of carbon content used in the carburisation experiment. More so, Fig. 10 also depict the microstructure of the carburised sample at 900 °C at ninety (90) minutes. The surface was observed to have an increased precipitate of carbon, the same characteristics was observed in Figs. 11–12 for samples carburised at 120 and 150 min respectively. However, at 180 min (Fig. 13), the metal surface was characterised with graphite precipitates. Thus, the presence of graphite indicates that diffusion actually took place during the carburisation process.

### Microhardness

The initial micro-hardness of the as-received cast iron was 288.41HV, which increased to 355.8 HV after carburisation at 180 min. This showed that there was an increase of about 67.39HV compared to the initial hardness value. This increment is a function of the adjustment in the characteristic parameters of the carburised material. Furthermore, the fatigue and wear resistance of grey cast iron component has been improved.

## Conclusions

From the study, it was established that atoms of the carbon used during carburisation moved in different fashion to eliminate concentration differences and eventually produce a homogeneous deposit on the grey cast iron material. These deposits are of varying composition. Thus, the understanding of atomic movements during the diffusion process has been established. More so, it was established that a diffusing atom of the carbon squeezed past the other surrounding atoms until it is deposited on the material. This process requires energy supply to force the atom to be deposited. This the reason for the temperature of carburisation.

In addition, the presence of graphite in the microstructures as observed in the carburised samples would help in the improvement of wear resistance because graphite presence serves as lubricant to the material. Thus, thermal fatigue is reduced. More so, increase in microhardness will help in reduction of surface abrasion of the grey cast iron during application.

Furthermore, Fick’s second law had been used to establish the non-steady state diffusion of carbon atoms into the substrate material (grey cast iron). However, a consequence of Fick’s second law is the possibility of achieving the constant composition profile for varying conditions, provided Dt remain constant. For several heat treatment and for most industrial applications, it will be possible to determine the temperature effect at varying time.

## Data Availability

All data generated or analysed during this study are included in this published article.

## List of symbols

*T*_*G*_: Temperature distribution in the grey cast iron sample
*x*_*G*_: X-coordinate in the grey cast iron sample
*y*_*G*_: Y-coordinate in the grey cast iron sample
*z*_*G*_: Z-coordinate in the grey cast iron sample
$d_{{x_{G} }} ,d_{{y_{G} }} ,d_{{z_{G} }}$: Infinitesimal control volume in the x, y and z-coordinate in the grey cast iron sample
$Q_{{x_{G} }} ,Q_{{y_{G} }} ,Q_{{z_{G} }}$: Rate of heat conduction perpendicular to the coordinates
$\dot{E}_{{g_{G} }}$: Thermal energy generated in the grey cast iron sample
$E_{{G_{st} }}$: Thermal energy stored by grey cast iron
$Q_{{x_{G} }} ,Q_{{y_{G} }} ,Q_{{z_{G} }}$: Conduction heat rate in various directions
*α*: Thermal diffusivity
c_0_: The carbon content for the as-received grey cast iron
c_s_: Assumed value from iron-carbon alloy system diagram between pure iron an interstitial compound, iron carbide (Fe_3_C)
c_x_: The concentration of the diffused carbon at a depth denoted by xn millimetre below the material surface at time t
erf: Error function from Fick’s second law
t: Time of carburisation in seconds
x: The depth (mm)

## Code availability

No code was used for the computation of the data reported in this study.
